# A study of peri-implant tissue clinical parameters in patients starting anti-osteoporosis medication after existing implant function: a prospective cohort study

**DOI:** 10.1186/s40729-024-00569-4

**Published:** 2024-11-05

**Authors:** Keisuke Seki, Takaaki Tamagawa, Hiroyasu Yasuda, Soichiro Manaka, Daisuke Akita, Atsushi Kamimoto, Yoshiyuki Hagiwara

**Affiliations:** 1 Implant Dentistry, Nihon University School of Dentistry Dental Hospital, Tokyo, Japan; 2 Department of Comprehensive Dentistry and Clinical Education, Nihon University School of Dentistry, Tokyo, Japan; 3 Department of Oral and Maxillofacial Surgery II, Nihon University School of Dentistry, Tokyo, Japan; 4 Department of Partial Denture Prosthodontics, Nihon University School of Dentistry, Tokyo, Japan; 5 Department of Periodontology, Nihon University School of Dentistry, Tokyo, Japan

**Keywords:** Anti-osteoporosis medications, Existing implant-triggered osteonecrosis, Osteoporosis, Peri-implantitis, Bisphosphonate, MRONJ

## Abstract

**Purpose:**

Recently, rare cases of medication-related peri-implant osteonecrosis of the jaw (PI-MRONJ) have been reported. In patients with functional implants who begin using anti-osteoporosis medications (AOMs) after implantation, PI-MRONJ is unpredictable and poses a significant threat to the patient. In this study, we aimed to evaluate the impact of AOMs on peri-implant tissues and to examine risk factors for peri-implantitis, a presumed trigger for PI-MRONJ.

**Methods:**

The study cohort consisted of patients who underwent implant maintenance treatment between January 2016 and February 2024. Patients were divided into AOM users (AOM group) and controls (control group). Clinical parameters were statistically evaluated, including implant probing depth (iPPD), implant bleeding on probing (iBoP), marginal bone resorption (MBL), and mandibular cortical index (MCI) measured at baseline and at the last visit. Risk factors were examined by multivariate analysis for adjusted odds ratios.

**Results:**

A total of 94 patients (35 male, 59 female) with 270 implants were recruited. The AOM group had 93 implants (24 patients). Comparison of clinical parameters showed significantly greater changes in iBoP (*p* = 0.000768) and MBL (*p* = 0.000863) scores over time in the AOM group than in the control group. Risk factors for peri-implantitis were a history of moderate or severe periodontal disease (OR: 15.8, 95% CI 3.6–69.3, *p* = 0.000256) and MCI class 3 (OR: 3.3, 95% CI 1.4–7.8, *p* = 0.00534).

**Conclusions:**

In implant treatment of AOM users, special attention should be paid to local inflammation, which is presumed to be the cause of PI-MRONJ.

**Graphical Abstract:**

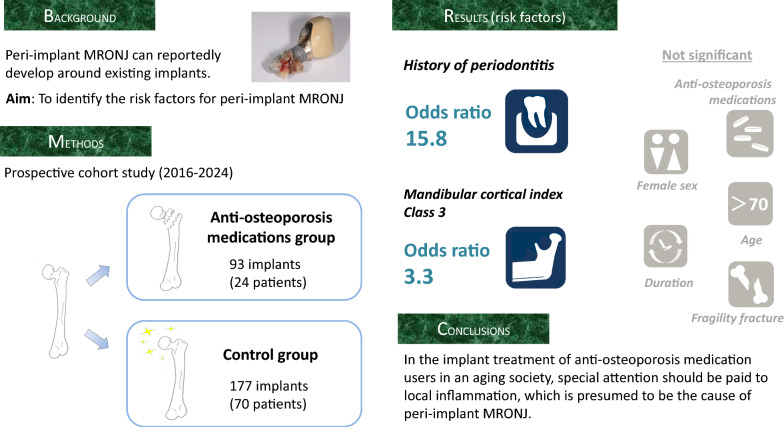

## Background

Among the various anti-osteoporosis medications (AOMs) used to treat osteoporosis and malignancy, antiresorptive agents (ARAs) such as bisphosphonate (BP) and denosumab (Dmab) are highly effective in preventing fragility fractures. These agents significantly reduce the relative risk of fractures and are effective in reducing their occurrence [1]. However, refractory osteonecrosis of the jaw has been reported to occur in long-term users of ARAs [2]. The severity of this rare condition, termed medication-related osteonecrosis of the jaw (MRONJ) [3], greatly reduces quality of life. The incidence of MRONJ is expected to increase globally as aging societies become more prevalent, and it is considered a problematic disease. The pathogenesis and treatment of MRONJ remain unclear, and the accumulation of evidence is still in its infancy. Position papers on MRONJ have been revised and updated [4], and consensus reports have been published worldwide [5, 6]. Although invasive surgical procedures such as tooth extractions and untreated inflammatory lesions have been implicated as major etiologic factors of this disease, information related to implant therapy is limited. Previous reports on MRONJ and implants have focused mostly on the safety of invasive implant placement procedures and their impact on osseointegration [7–11]. In particular, recent studies have considered existing implants themselves as a risk [12, 13], and this is an area that should be clarified as soon as possible.

In recent years, implant therapy has become the gold standard for predictable and reliable prosthetic treatment [14]. Although long-term clinical success is a common outcome, implant treatment is not without risks and problems. Systemic diseases and medications that are increasingly used by aging patients have an effect on the local oral environment [15, 16]. Significantly, we have previously reported cases of MRONJ in patients who started using AOMs during long-term stable implant function rather than before implant placement [17–19]. The impact of the use of AOMs on existing functioning peri-implant tissues has not yet been studied, and the scientific information on the relationship between local inflammation around implants and the development of MRONJ is still inconsistent and incomplete. Contemporary implantologists need to deepen their knowledge in these areas and fill the evidence gap. Clinicians must elevate the implant treatment of complex symptomatic patients so that it becomes more predictable.

The purpose of this prospective cohort study was to examine the impact of AOM use on the peri-implant tissues of existing functioning implants. We hypothesized that peri-implantitis is a surrogate endpoint and that the implant probing pocket depth, implant bleeding on probing, and marginal bone loss can be used as the basis for its diagnosis. The patient/population, intervention, comparison and outcomes (PICO) for this study was as follows. P: patients with a functioning implant, I: patients who have begun using AOMs, C: comparison with healthy patients, O: observation of changes in the clinical parameters of peri-implant tissues. The ultimate goal of this observational study was to determine whether existing peri-implant tissue inflammation is an independent etiological factor for MRONJ.

## Methods

This prospective cohort study was approved by the Ethics Committee of Nihon University School of Dentistry (Permit No. EP16D013) and was conducted in accordance with the 1975 Declaration of Helsinki as revised in 2013, as well as in accordance with the guidelines for observational/descriptive studies on enhanced reporting of observational studies in epidemiology [20]. The cohort of this study comprised patients who attended the Department of Dental Implantology at Nihon University Dental Hospital for maintenance treatment between January 2016 and February 2024.

### Case selection criteria

**Fig. 1:**
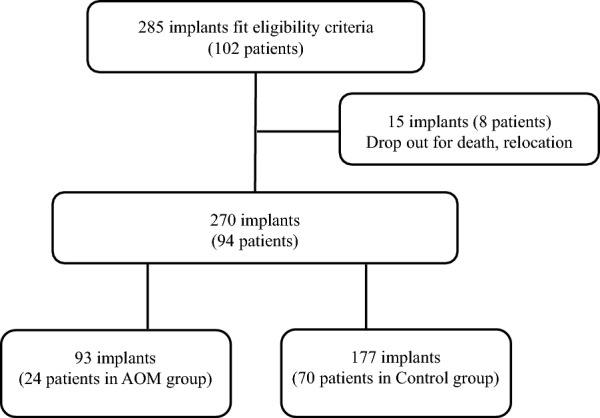
Flowchart of the study population

**Fig. 2:**
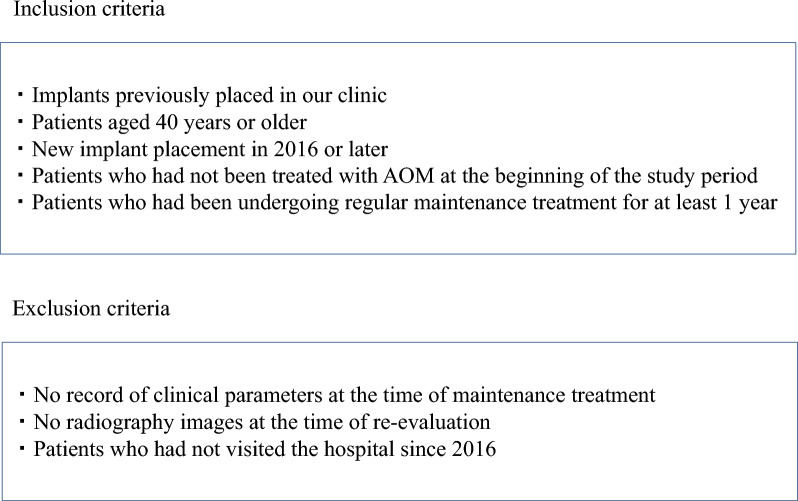
Inclusion and exclusion criteria

To avoid interoperator bias, a single periodontist (KS) diagnosed and planned the treatment of all patients. Patients were provided with periodontal treatment, implant surgery, prosthetic treatment, and maintenance as necessary. The inclusion criteria were as follows: (1) implants previously placed in our clinic, (2) patients aged 40 years or older who visited the clinic for implant maintenance or new implant placement in 2016 or later when the study was initiated, (3) patients who had not been treated with AOMs at the beginning of the study period, (4) patients who had been undergoing regular maintenance treatment for at least 1 year after the placement of the superstructure. Exclusion criteria were: (1) no record of clinical parameters at the time of maintenance treatment, (2) no dental X-ray or digital panoramic radiography (DPR) images at the time of re-evaluation, (3) patients who had not visited the hospital since 2016 (for reasons including death and relocation) (Figs. 1 and 2).

### Implant placement protocols and prosthetic design

All implant surgeries were performed with a two-staged approach and according to the manufacturer’s instructions under either local anesthesia alone or with intravenous sedation. For implant sites with insufficient bone mass, simultaneous bone grafting with autogenous or artificial bone was performed at the time of implant placement. Postoperative medications were oral antibiotics (cefuroxime axetil, cefcapene pivoxil hydrochloride hydrate, and amoxicillin hydrate) three times a day for 3 days after surgery to prevent infection. Analgesics (diclofenac sodium and loxoprofen sodium hydrate) were prescribed as needed. Seven days after each surgery, all sutures were removed, and the patients were instructed to maintain good oral hygiene. After a sufficient period of soft tissue healing, the superstructure was fabricated. In the past, gold alloy custom abutments were combined with metal-ceramic restorations, but in recent years, titanium bases or titanium abutments with zirconia crowns have been used. The type of prosthetic fixation was determined by the direction of implant placement, the location of access holes, esthetics and cleanability, and whether fixation was by screw or cement.

### Investigation of clinical parameters of peri-implant tissue

The baseline for recording the clinical parameters of the peri-implant tissues was at the start of maintenance after placement of the final prosthesis. However, for the cases in which prosthetic treatment had been completed at our clinic and maintenance had already started, we used the examination data from the earliest visit within the study period. At the time of re-evaluation, data from the last visit within the study period were evaluated. The clinical parameters measured for each individual implant are set out below. Data collection and analysis were performed by two operators. Statistical analysis of the final dataset was then performed by a secondary operator, who remained blind to the study objectives and methods.

#### Implant probing pocket depth

To standardize the probing pressure (0.15 N), the operator's measured pressure was calibrated many times using an electronic scale. The operator inserted a periodontal pocket probe (11 Colorvue^®^ Probe Kit, Hu-Friedy, Chicago, IL, USA) into the peri-implant pocket with a force of 0.15 N and measured the depth in 1-mm increments at six points. The mean depth and the deepest point were calculated from the six pocket depths. The implant probing pocket depth (iPPD) was calculated as the difference in depth between the baseline and the last visit (last visit minus baseline).

#### Implant bleeding on probing

Implant bleeding on probing (iBoP) was evaluated 10 s after the six-point probing. No bleeding was quantified as negative (score: 0) and bleeding as positive (score: 1), and the mean value of the six points was calculated (final score range: 0–1). The iBoP was calculated as the difference between the baseline value and the value at the last visit (last visit minus baseline).

#### Evaluation of bone resorption over time on dental X-ray

**Fig. 3:**
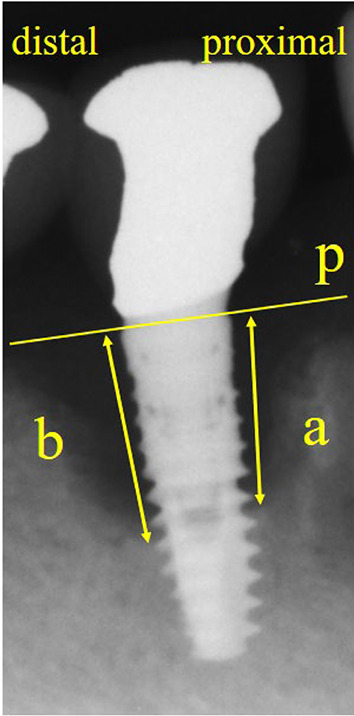
The amount of marginal bone loss was defined as the average of a and b. a: distance from the proximal platform of the bottom of the bone defect (mm), b: distance from the distal platform of the bottom of the bone defect (mm), p: implant platform

The amount of marginal bone loss (MBL) around the implants was measured on dental radiographs taken using a paralleling technique (irradiated at 110 kV, 1–20 mA, and effective dose of 100 µSv). The distance from the proximal or distal platform of the implants to the bottom of the bone defect was measured on the obtained images (TechM@trix, SDS Viewer, Tokyo, Japan), and the amount of bone resorption per implant was averaged (Fig. 3). The MBL was calculated as the difference between the baseline value and the value at the last visit (last visit minus baseline).

### Diagnosis of peri-implantitis

After the superstructure was placed, maintenance treatment was performed at intervals of 3–6 months according to the oral hygiene of each patient. Peri-implant disease was diagnosed by probing and radiographic examination of the peri-implant tissues during the subsequent follow-up re-evaluation. Peri-implantitis was diagnosed in accordance with the statement of the World Workshop on the Classification of Periodontal and Peri-Implant Diseases and Conditions, considering inflammation of the peri-implant mucosa and progressive resorption of the supporting bone [21] (i.e., a deepened implant probing depth or radiographic bone resorption compared with baseline). Additional diagnostic criteria were: (1) probing depth greater than 6 mm, (2) drainage or bleeding during probing, and (3) radiographic evidence of bone resorption greater than 25% of the implant length.

### Diagnosis of PI-MRONJ

PI-MRONJ was diagnosed when MRONJ developed in the tissue surrounding the implant. Patients were diagnosed with MRONJ when they had a history of taking bone resorption inhibitors or angiogenesis inhibitors, no history of radiation therapy of the jawbone, and bone exposure or osteonecrosis in the oral or maxillofacial region lasting more than 8 weeks [4]. Staging was classified as 0–3; stage 0 was defined as clinical symptoms persisting for at least 8 weeks without bone exposure, or bone exposure and persistence of symptoms for at least 4 weeks, and nonspecific clinical or radiological changes or symptoms without osteonecrosis.

### Mandibular cortical index assessment

A blurred and rough appearance of the mandibular inferior cortical bone on DPR images has been reported to correlate with the severity of osteoporosis [22]. Using all DPR sample images, the mandibular inferior cortical bone morphology was classified into three types, according to the report of Klemetti et al. [23]. Class 1 has a smooth cortical bone inner surface, class 2 has an irregular inner surface of cortical bone with linear resorption, and class 3 has severe linear resorption and cortical bone rupture over the entire cortical bone. Two sites were evaluated per patient, one on the left side and one on the right side, and each was recorded. The mandibular cortical index (MCI) was assessed twice by a dentist (KS) who was trained in classification beforehand, and the second assessment was used. The higher MCI score (whether right or left side) was adopted as the representative value, and was used as the final MCI evaluation for each patient.

### Data source and group settings

Information on age, sex, body mass index (BMI), history of fragility fracture (excluding traumatic injuries caused by sports or accidents before adolescence) [24, 25], smoking habits, and history of moderate or severe periodontitis was collected from the initial examination records. From the records collected during the maintenance treatment, information was extracted on AOM treatment initiated after implant function, implant size and placement site, type of superstructure fixation (cement or screw fixation, including side screws), MCI classification, peri-implant clinical parameters (iPPD, iBoP, MBL), peri-implantitis, and MRONJ development. For all implants, the period from the day the superstructure was placed to the last visit was set as the observation period. The clinical parameters of implants removed as a result of peri-implantitis were taken from the data recorded most recently at the time of removal. Patients were divided into two groups: the AOM group (those who started AOM treatment after implant function) and the control group (those with implants who did not undergo AOM treatment).

### Statistical analysis

All statistical analyses were performed using EZR (Saitama Medical Center, Jichi Medical University, Japan), a graphical user interface of R (The R Foundation for Statistical Computing, Vienna, Austria, version 4.0.0) [26]. EZR is customized for easy analysis by incorporating R Commander, a package of additional functions of R. The software is designed to add statistical functions frequently used in biostatistics.

The required sample size calculated with a two-tailed test with an alpha error of 0.05 and power of 0.8 was 37 for the AOM group and 74 for the control group. Using the demographic data of the AOM and control groups obtained by descriptive statistics, we tested the statistical differences for each variable. Normality of the data distribution for continuous variable outcomes (age, BMI, follow-up period, iPPD, iBoP, and MBL) was determined by the Kolmogorov–Smirnov test, and P ≥ 0.05 was considered as a normal distribution. When the data followed equal variances and were normally distributed, Student’s t test was performed. When they did not follow a normal distribution, the Mann–Whitney U test was performed. Fisher's exact test or a chi-square test was employed for categorical variables (sex, history of fragility fracture, smoking habit, history of periodontitis, MCI classification, presence of peri-implantitis, and type of superstructure fixation).

**Fig. 4:**
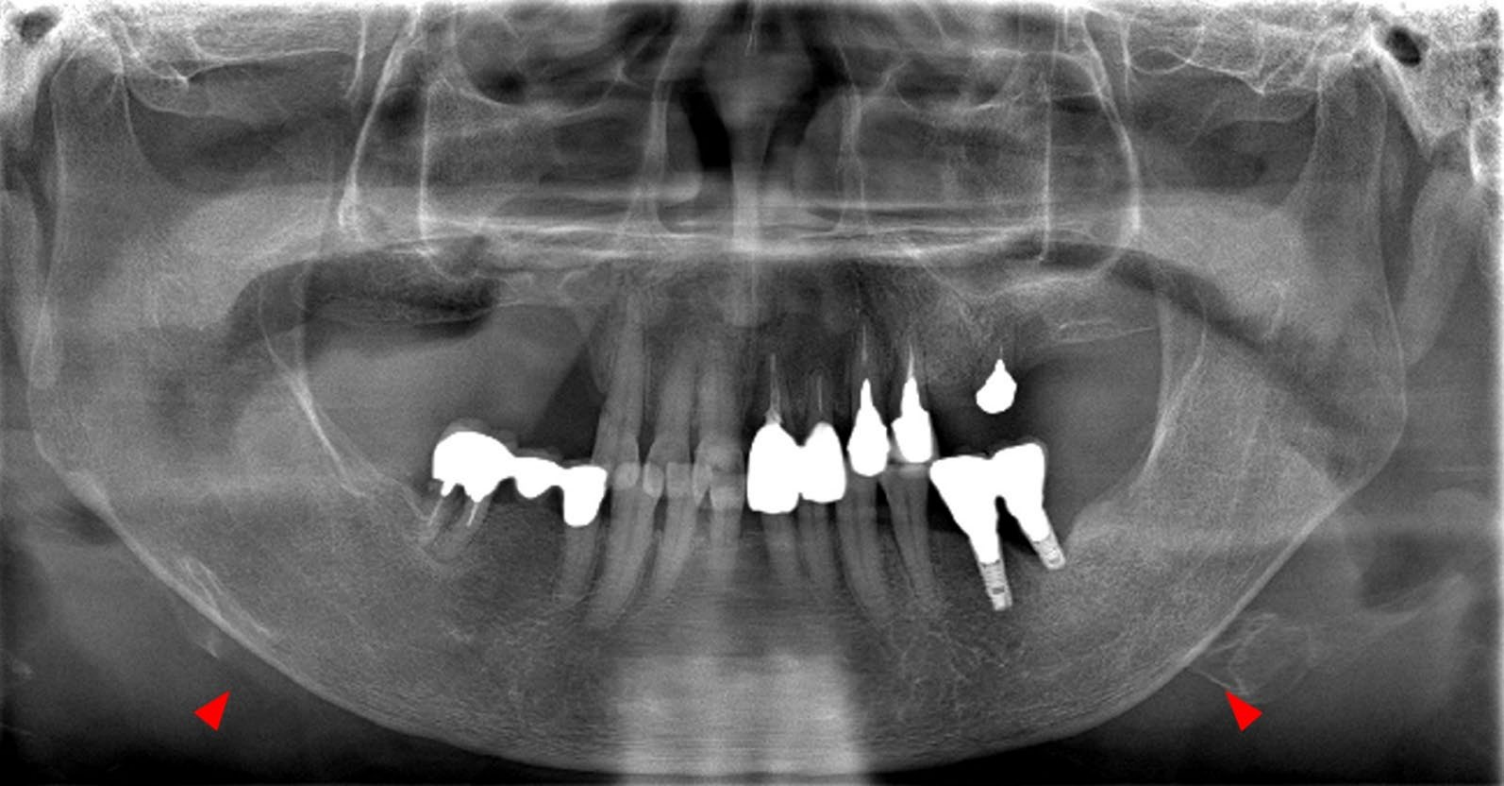
Digital panoramic radiography imaging findings in case 5. A 70-year-old woman had been using alendronate orally for 4 years after implant function. The #16–#14 implants were removed as a result of peri-implant medication-related osteonecrosis of the jaw (PI-MRONJ), and it has been 7 years since the drug was changed to raloxifene hydrochloride (SERM). The #36 implant was diagnosed with peri-implantitis. The mandibular cortical index (MCI) classification was class 3 (arrowhead)

To examine risk factors, univariate analysis was first performed using the items that differed between the two groups as explanatory variables and peri-implantitis as the objective variable, and crude odds ratios (ORs) and 95% confidence intervals (CIs) were calculated. A logistic regression model was used to measure the association between predictor and outcome variables while controlling for confounding factors, and adjusted odds ratios (aOR) were determined. P < 0.05 was considered to indicate a statistically significant difference. Even when multiple implants were present in a single patient, the health status of the host was expected to change because of the different timing of implant placement, prosthetic methods, and functional periods. Hence, for the purpose of examining the effect of AOM treatment on the clinical parameters of individual implants, the tests were analyzed on an implant basis (Fig. 4).

## Results

### Demographic data

Patient demographics are summarized in Table 1. In this prospective cohort study, after 8 patients (with 15 implants) dropped out, 94 patients (59 women and 35 men) with 270 implants were included in the final study. The mean age of the patients was 69.8 ± 9.7 (range 43–88) years overall, which was significantly higher in the AOM group (76.7 ± 6.3) than in the control group. AOM users after implant function totaled 24 (25.5%), 21 women and 3 men. Twelve patients (12.8%), all of whom were women, had a history of fragility fracture in both groups, with significantly more in the AOM group (only 3 in the control group). Overall, 27 (28.7%) had a smoking habit that included a history of past smoking, and there were no significant differences between groups. The mean BMI was 22.3 ± 2.9 (range 17.4–29.4) overall, and 17 patients (18.1%) had a BMI greater than 25; however, there were no significant differences between the groups. Forty-seven (50%) of all patients had a history of periodontitis, and there was no significant difference between the two groups. MCI was class 1 in 22 (23.4%), class 2 in 50 (53.2%), and class 3 in 22 (23.4%) patients. By subgroup, class 1 was significantly more common in the control group (31.4% of the group) as was class 3 in the AOM group (45.8% of the group). The most commonly used drugs within the AOM group were BPs (19 cases), followed by vitamin D3 (15 cases); selective estrogen receptor modulators (SERMs) were used in 6 cases, teriparatide in 3 cases, and denosumab and methotrexate in 2 cases each (Fig. 1). The mean duration of use was 3.6 years (median 1–16 years). Details of the AOM group are shown in Table 2.

**Table 1:** Demographic and patient characteristics of the AOM and the control groups

	AOM (*n* = 24)	Control (*n* = 70)	*P* value	Significant difference
^a^Sex				
Female	21 (87.5)	38 (54.3)	0.004	**
^c^Age (years)	76.7 ± 6.3	67.4 ± 9.5	0.000	**
^c^Body mass index (kg/m^2^)	21.7 ± 2.6	22.6 ± 3.0	0.189	ns
^a^History of fragility fracture	9 (37.5)	3 (4.3)	0.000	**
^a^Smoking	5 (20.8)	22 (31.4)	0.322	ns
^a^History of periodontitis (moderate to severe)	17 (70.8)	30 (42.8)	0.033	**
Mandibular cortical index				
^b^Class 1	0 (0)	22 (31.4)	0.001	**
^a^Class 2	13 (54.2)	37 (52.0)	0.912	ns
^a^Class 3	11 (45.8)	11 (15.7)	0.003	**
Antiresorptive therapy *overlapping				
Bisphosphonate (oral 18, injection 1)	19			
Denosumab	2			
Tocilizumab	1			
Teriparatide	3			
Selective estrogen receptor modulator	6			
Vitamin D3	15			
Calcium L-aspartate hydrate	1			
Methotrexate	2			
Duration of antiresorptive therapy (years)	3.6 ± 2.7 (1–16)			
1–3 years	26			
Over 4 years	23			

Mean ± S.D., or *n* (%)

^a^Chi-square test

^b^Fisher's exact test

^c^Student's t-test

*AOM* anti-osteoporosis medication, *ns* not significant

^**^P < 0.01

**Table 2:** Medication data for the AOM group

Case	Age	Sex	Primary disease	Medication	Type	Interval	Duration	Number of	Type of MRONJ	Severity
							(Years)	Existing implants		
1	73	Male	Osteoporosis, prostate cancer	Denosumab	Subcutaneous injection	6 months	16	4		
2	72	Female	Osteoporosis	Raloxifene hydrochloride	Oral	Daily	4	7		
				Alfacalcidol (VD3)	Oral	Daily	4	7		
3	86	Female	Osteoporosis	Alendronate	Oral	Daily	4	4		
				Eldecalcitol (VD3)	Oral	Daily	4	4		
				Teriparatide	Subcutaneous injection	1 week	2	4		
4	72	Female	Osteoporosis, rheumatoid arthritis	Eldecalcitol (VD3)	Oral	Daily	4	2		
				Methotrexate	Oral	A few days	4	2		
5	61	Female	Osteoporosis, breast cancer	Alendronate	Oral	Daily	5	1		
				Eldecalcitol (VD3)	Oral	Daily	5	1		
6	79	Female	Osteoporosis, thyroid cancer	Alendronate	Oral	1 week	4	4	PI-MRONJ	(Stage 2)
				Raloxifene hydrochloride	Oral	Daily	7	4		
7	79	Female	Osteoporosis	Minodronate	Oral	4 weeks	8	3		
				Teriparatide	Subcutaneous injection	1 week	2	3		
8	73	Female	Osteoporosis	Teriparatide	Subcutaneous injection	3 days	2	8		
				Alfacalcidol (VD3)	Oral	Daily	2	8		
9	75	Female	Osteoporosis	Minodronate	Oral	4 weeks	2	9		
				Alfacalcidol (VD3)	Oral	Daily	2	1		
				Alfacalcidol (VD3)	Oral	Daily	2	1		
10	76	Female	Osteoporosis	Bazedoxifene Acetate	Oral	Daily	5	3		
				Eldecalcitol (VD3)	Oral	Daily	7	3		
				Alendronate	Oral	Daily	2	3		
11	80	Female	Osteoporosis	Minodronate	Oral	4 weeks	11	9		
12	82	Female	Osteoporosis	Minodronate	Oral	4 weeks	4	2		
13	73	Female	Osteoporosis	Alendronate	Oral	1 week	1	6		
14	85	Male	Osteoporosis, renal pelvis cancer	Unknown BPs	Oral	Daily	4	2		
				Unknown VD3	Oral	Daily	4	2		
15	86	Female	Osteoporosis	Unknown BPs	Oral	Daily	2	2		
16	72	Female	Osteoporosis, rheumatoid arthritis	Risedronate	Oral	Daily	2	2	MRONJ	(Stage 2)
				Eldecalcitol (VD3)	Oral	Daily	2	2		
				Ibandronate	Oral	Daily	3	2		
				Tocilizumab	Subcutaneous injection	1 week	4	2		
				Methotrexate	Oral	1 week	3	2		
17	83	Female	Osteoporosis	Alendronate	Oral	1 week	1	3		
				Eldecalcitol (VD3)	Oral	Daily	3	3		
				Bazedoxifene acetate	Oral	Daily	1	3		
18	80	Female	Osteoporosis	Eldecalcitol (VD3)	Oral	Daily	2	3		
				Raloxifene hydrochloride	Oral	Daily	2	3		
19	87	Male	Osteoporosis	Minodronate	Oral	Daily	2	3	PI-MRONJ	(Stage 2)
				Eldecalcitol (VD3)	Oral	Daily	2	3		
				Calcium L-aspartate hydrate	Oral	Daily	2	3		
				Denosumab	Subcutaneous injection	6 months	2	3		
20	72	Female	Osteoporosis	Ibandronate	Subcutaneous injection	1 month	7	3		
				Eldecalcitol (VD3)	Oral	Daily	5	3		
				Raloxifene hydrochloride	Oral	Daily	5	3		
21	72	Female	Osteoporosis	Alendronate	Oral	Daily	2	1		
22	71	Female	Osteoporosis	Eldecalcitol (VD3)	Oral	Daily	1	5		
23	72	Female	Osteoporosis, stomach cancer	Risedronate	Oral	1 week	2	1		
24	79	Female	Osteoporosis	Risedronate	Oral	1 week	1	12		

*AOM* anti-osteoporosis medication, *MRONJ* medication-related osteonecrosis of the jaw, *PI-MRONJ* peri-implant MRONJ, *VD3* vitamin D3

Information at the implant level for each group is shown in Table 3. The total number of eligible implants placed was 270, with 134 implants in the maxilla and 136 implants in the mandible, almost equal numbers. The most common implants used were Nobel Biocare (139), Astra Tech Implant (40), Straumann (39), Biomet 3i (28), and other types with fewer than 6 implants of each type (STERI-OSS, AQB, Endopore, GC, KYOCERA), all with a rough surface structure. There was no significant difference between the two groups in the type of fixation of the superstructure (screw or cement fixation). The overall mean observation period of implants placed was 11.8 ± 5.8 years, which was significantly longer in the AOM group (14.2 years) than in the control group. The overall prevalence of peri-implantitis was 17.0% (46 of 270 implants) at the implant level and 23.4% (22 of 94 patients) at the patient level, and was significantly higher in the AOM group (26.9%, implant level). Twenty-two implants (8.1%) failed and were removed during the study period. The reasons for failure were advanced peri-implantitis in 21 implants and overload in one. Peri-implant MRONJ occurred in two patients in the AOM group (4 implants in total, all in Stage 2).

**Table 3:** Implant characteristics for the AOM group and the control group

	AOM (*n* = 93)	Control (*n* = 177)	*P* value	Significant difference
^a^Implant site				
Maxilla	46 (49.4)	88 (49.7)	1.000	ns
Mandible	47 (50.5)	89 (50.2)		
^a^Peri-implantitis	25 (26.9)	21 (11.9)	0.002	**
^b^Peri-implant MRONJ	4 (4.3)	0 (0)	0.014	*
^a^Superstructure				
Cement retention	43 (46.2)	63 (35.6)	0.116	ns
Screw retention (include side-screw system)	50 (53.8)	114 (64.4)		
^c^Mean maintenance duration (years)	14.2 ± 6.5	11.0 ± 5.3	0.002	*
(Range, median)	(3–26, 15.5)	(1–20, 11.5)		

Mean ± S.D., *n* (%)

^a^Chi-square test

^b^Fisher's exact test

^c^Student's t-test

*AOM* antiosteoporosis medication, *MRONJ* medication-related osteonecrosis of the jaw, *ns* not significant

^*^P < 0.05, **P < 0.01

### Implant-based data evaluation

Clinical parameters of peri-implant tissues are shown in Table 4. At baseline, the mean iPPD in the AOM group (3.2 ± 1.2 mm) was significantly greater than that in the control group. Similarly, at the last visit, the mean iPPD (4.0 ± 2.2 mm) and the deepest iPPD (4.9 ± 2.5 mm) and iBoP score (0.3 ± 0.4) were significantly greater in the AOM group. The difference between the last visit and baseline values was significantly greater in the AOM group than in the control group for iBoP score (0.2 ± 0.4) and MBL (2.0 ± 6.4 mm).

**Table 4:** Clinical parameters for the AOM and control groups

	Baseline				Last visit			
	iPPD (mm)		iBoP (0–1)	MBL (mm)	iPPD (mm)		iBoP (0–1)	MBL (mm)
	Mean	Maximum			Mean	Maximum		
AOM group	3.2 ± 1.2	3.8 ± 1.7	0.2 ± 0.2	1.4 ± 1.1	4.0 ± 2.2	4.9 ± 2.5	0.3 ± 0.4	2.4 ± 2.6
(Range, median)	(0–11.0, 3)	(0–12.0, 3)	(0–1, 0)	(0–5.0, 1.2)	(2.0–11.0, 3.2)	(2.0–11.0, 4)	(0–1, 0.2)	(0–11.0, 1.7)
Control group	2.9 ± 0.5	3.5 ± 0.9	0.2 ± 0.2	1.4 ± 1.2	3.4 ± 1.8	4.1 ± 2.0	0.2 ± 0.3	2.2 ± 2.5
(Range, median)	(1.8–6.7, 3)	(2.0–8.0, 3)	(0–1, 0.2)	(0–9.0, 1.2)	(0–13.0, 3)	(2.0–13.0, 4)	(0–1, 0)	(0–13.0, 1.5)
Significant difference	*	ns	ns	ns	*	*	**	ns

Mean ± S.D., Mann–Whitney U test

*AOM* antiosteoporosis medication, *iPPD* implant probing depth, *iBoP* implant bleeding on probing, *MBL* marginal bone resorption, *ns* not significant

^*^P < 0.05, **P < 0.01

In univariate and multivariate analyses, cutoff values were set for explanatory variables that were continuous variables. The cutoff value was assumed to be the median of the means of the AOM and control groups, respectively. Specifically, age was defined as 1 for 70 years or older and 0 for 69 years or younger, and the observation period was defined as 1 for 12 years or longer and 0 for 11 years or less. Univariate analysis with peri-implantitis as the objective variable revealed the following significant differences: use of AOMs (OR: 2.73), age > 70 years (OR: 2.97), history of fragility fracture (OR: 0.89), history of moderate or severe periodontitis (OR: 15.3), MCI class 3 (OR: 2.79), and 12-year or longer observation period (OR: 1.03). Additionally, multivariate analysis was performed to examine adjusted odds ratios. The results showed a significant difference in the prevalence of moderate or severe periodontitis (aOR: 15.80) and MCI class 3 (aOR: 3.34). No significant differences were found for the other independent variables (Table 5).

**Table 5:** Risk indicators for peri-implantitis according to logistic regression analysis

Explanatory variable	Unadjusted			Adjusted		
	Odds ratio	[95% CI]	P value	Odds ratio	[95% CI]	P value
AOM (yes; 1, no; 0)	2.73	[1.43–5.21]	**	1.85	[0.82–4.20]	ns
Sex (female; 1, male; 0)	1.28	[0.64–2.57]	ns	0.54	[0.21–1.39]	ns
Age (over 70; 1, others; 0)	2.97	[1.37–6.45]	**	2.02	[0.87–4.71]	ns
History of fragility fracture (yes; 1, no; 0)	0.89	[0.40–1.98]	**	0.47	[0.18–1.21]	ns
History of periodontitis (yes; 1, no; 1)	15.30	[3.63–64.80]	**	15.80	[3.60–69.3]	**
MCI (class 3; 1, others; 0)	2.79	[1.46–5.33]	**	3.34	[1.43–7.81]	**
Observational duration (over 12 years; 1, others; 0)	1.03	[0.54–1.95]	**	1.24	[0.60–2.55]	ns

Adjusted for AOM use, sex, age, fracture, periodontitis, MCI, and duration of maintenance

*AOM* anti-osteoporosis medication, *CI* confidence interval, *MCI* mandibular cortical index, *ns* not significant

^**^P < 0.01

## Discussion

This single-center prospective cohort study observed changes in peri-implant tissue parameters in patients who started AOM treatment during implant function. Although the pathophysiological hypothesis of MRONJ development is multifactorial [4], in recent years, research has focused on the effects of persistent dental infection [27–29]. Inflammation in the oral cavity lowers pH and leads to the release and activation of bone-bound BP molecules [30, 31], which may have a negative effect on the jaw bone tissue. Some researchers have postulated that peri-implantitis is an independent risk factor for the development of MRONJ [32]. The present study focused on the clinical parameters of peri-implant tissue as surrogate markers of the local inflammatory response. The AOM group in this research was characterized by predominantly older female patients. The most commonly used drugs were third-generation oral BPs. Some researchers have noted that MRONJ can be severe, even with low doses of oral AOMs, so care should be taken not to underestimate their impact [33]. Evidence for BPs and Dmab as causative agents of MRONJ is available, while the risk of MRONJ with other drugs (methotrexate, tocilizumab, and SERMs) has not yet been established in large cohort studies, and the presence of a causal relationship is unclear. However, the occurrence of MRONJ near the natural root apex of one tocilizumab user was a characteristic finding in the present study [34]. The average duration of drug use in the AOM group was 3.6 years. Although the evidence for long-term drug use as a risk factor for MRONJ is weak [4], a recent scoping review reported an average of 5.7 years from the start of anti-angiogenic or anti-resorptive therapy to the onset of implant presence-triggered osteonecrosis [13]. This medical information signals that caution is required in long-term implant maintenance treatment. If only BP and Dmab users had been included, the present study might have provided further evidence that these drugs affect not only the development of MRONJ but also affect the development of PI-MRONJ. However, the effects of BP and Dmab alone are currently unknown because the present study also included patients who were taking drugs that have not yet been identified as suspected of causing MRONJ. Future studies should investigate drug categories (e.g., tocilizumab, teriparatide, SERM, vitamin D3, calcium L-aspartate hydrate, and methotrexate) that could potentially cause MRONJ but have not yet been evaluated in this aspect. Studies of MCI classification have shown a close correlation between class 3 and osteoporosis. In a previous study focusing on DPR imaging findings, we found that the use of AOMs was not associated with increased calcification of the mandibular inferior cortical bone [35]. Similarly, the imaging findings in the present study were consistent with those reported in previous studies, with many cases showing an MCI class 3 phenotype in the AOM group.

The prevalence of peri-implantitis within the AOM group was greater than previously reported [36], which is a notable finding. Comparing changes in peri-implant clinical parameters as signs of worsening inflammation over time, iBoP and MBL were significantly increased in the AOM group. This suggests that chronic inflammation in the peri-implant tissues of the AOM group may persist or worsen compared with the control group, which is a new finding. Interestingly, a histopathologic study of peri-implantitis that preceded PI-MRONJ reported bleeding and deep probing depth (7 ± 1.2 mm) around all implants [37]. However, it is significant that the present study further evaluated the condition in the pre-developmental stage of the lesion. A previous systematic review suggested that successful implant treatment in AOM users depends on maintaining healthy peri-implant tissues with regular observation for 3 years after the start of bone resorption control treatment [38]. Although there have been reports of chemotherapeutic agents damaging the implant interface [39], no studies have directly shown that AOM use induces local inflammation of peri-implant tissue. It remains unclear whether the interacting effects of the various components of complex oncologic treatment regimens contribute to peri-implantitis or MRONJ development. Some animal studies have shown that the development of peri-implantitis lesions is unlikely to be accelerated by bone resorption inhibitors or anti-angiogenic agents [40], and these associations should be clarified in future studies.

Recent reviews examining the safety of implant placement in ARA users have stated that the success rate is not problematic and that the risk of not achieving osseointegration is small [9, 11]. Another review suggested that successful implant treatment is possible, but that bone augmentation should be avoided and that perioperative antimicrobial prophylaxis is strongly recommended [41]. However, in the present study, as a secondary outcome of the study, we re-examined the factors affecting the development of peri-implantitis that influence the development of PI-MRONJ. Therefore, the inclusion of information related to osteoporosis in addition to the known risk of peri-implantitis is a new perspective. In examining etiology, the crude odds ratios were, in descending order, history of periodontitis (OR = 15.30), age (OR = 2.97), MCI class 3 (OR = 2.79), and AOM use (OR = 2.73). After adjusting for confounding factors, logistic regression analysis finally revealed a significant difference in the results for history of periodontitis (aOR = 15.80) and MCI class 3 (aOR = 3.34). Various animal studies have examined the effects of AOM use on periodontal tissues, and it has been reported that zoledronate disrupts the peri-implant environment, causing mild persistent inflammation and increasing the amount of inactive bone tissue [42]. In contrast, another report states that the development of peri-implantitis lesions is unlikely to be accelerated by anti-resorptive or anti-angiogenic agents [40], and there is no consensus on the possibility that bone resorption inhibitors may cause inflammation in the peri-implant tissue. Class 3 of the MCI classification is associated with more severe osteoporosis, as mentioned above. While evidence is building for a close association between the severity of periodontal disease and osteoporosis [43, 44], the association between peri-implant tissue condition and osteoporosis has only just begun to be studied. The findings of the present study will contribute to tailor-made treatment for each patient in the diagnosis and prognosis of future implant treatment.

The strength of this study lies in the rarity of having examined the clinical parameters of the peri-implant tissues of patients taking AOMs. Research related to osteoporosis patients and bone resorption inhibitors has been increasing in recent years, and the results of this study may be a basis for further research in implant dentistry. However, the single-center design of the study is a limitation, and the application of these results to general dental practice should be treated with caution. Future research should be undertaken with a larger sample size through a multicenter survey to enable translation of the results to improvements in clinical practice. Another study limitation is that the definition of the observation period needs to be improved. Using the period from superstructure installation to the last visit as the observation period may have overlooked inconsistent follow-up intervals. Future studies should standardize the follow-up intervals to improve the data continuity and the reliability of the analysis.

## Conclusions

We conducted a single-center prospective cohort study on the evaluation of the clinical parameters of peri-implant tissues in patients using AOMs. AOM users showed increased peri-implant tissue bleeding scores and greater marginal bone resorption, suggesting a persistent inflammatory state. Risk factors for peri-implantitis were identified, including a history of moderate to severe periodontitis and coarsening of the mandibular inferior cortical bone morphology. Implant treatment AOM users should be carefully monitored, with special attention paid to local inflammation, which is presumed to be the cause of PI-MRONJ. The clinical implication is that clinicians managing implant patients taking AOMs should not view peri-implantitis as a single inflammatory disease, but should suspect it as an early manifestation that precedes the development of PI-MRONJ. In addition, the present findings suggest that frequent follow-up visits may help to prevent the serious exacerbation of symptoms.

## Data Availability

No datasets were generated or analysed during the current study.

## Abbreviations

AOM: Anti-osteoporosis medication
ARA: Antiresorptive agent
BoP: Bleeding on probing
BP: Bisphosphonate
CI: Confidence interval
Dmab: Denosumab
iBoP: Implant bleeding on probing
iPPD: Implant probing pocket depth
MBL: Marginal bone loss
MCI: Mandibular cortical index
MRONJ: Medication-related osteonecrosis of the jaw
PI-MRONJ: Peri-implant medication-related osteonecrosis of the jaw
SERM: Selective estrogen receptor modulators
